# Histopathological evaluation of abdominal aortic aneurysms with deep learning

**DOI:** 10.1186/s13000-025-01684-5

**Published:** 2025-09-16

**Authors:** Fiona R. Kolbinger, Omar S. M. El Nahhas, Maja Carina Nackenhorst, Christine Brostjan, Wolf Eilenberg, Albert Busch, Jakob Nikolas Kather

**Affiliations:** 1 Department of Visceral, Thoracic and Vascular Surgery, Faculty of Medicine, University Hospital, Carl Gustav Carus, TUD Dresden University of Technology, Fetscherstraße 74, 01307 Dresden, Germany; 2 Else Kröner Fresenius Center for Digital Health (EKFZ), TUD Dresden University of Technology, Fetscherstraße 74, 01307 Dresden, Germany; 3 Weldon School of Biomedical Engineering, Purdue University, West Lafayette, IN USA; 4 Department of Pathology, Medical University of Vienna, Vienna, Austria; 5 Division of Vascular Surgery, Department of General Surgery, Medical University of Vienna and Vienna General Hospital, Vienna, Austria; 6 Department of Medicine I, University Hospital Dresden, Dresden, Germany; 7 Medical Oncology, National Center for Tumor Diseases (NCT), University Hospital Heidelberg, Heidelberg, Germany

**Keywords:** Abdominal aortic aneurysm, Computational pathology, Vascular pathology, Deep learning

## Abstract

Computational analysis of histopathological specimens holds promise in identifying biomarkers, elucidating disease mechanisms, and streamlining clinical diagnosis. However, the application of deep learning techniques in vascular pathology remains underexplored. Here, we present a comprehensive evaluation of deep learning-based approaches to analyze digital whole-slide images of abdominal aortic aneurysm samples from 369 patients from three European centers. Deep learning demonstrated robust performance in predicting inflammatory characteristics, particularly in the adventitia, as well as fibrosis grade and remaining elastic fibers in the tunica media from Hematoxylin and Eosin (HE)-stained slides (mean AUC > 0.70 in two external test cohorts). Models trained on Elastica van Gieson (EvG)-stained slides overall performed similar to models trained on HE-stained WSI for detection of calcification and fibrosis. For prediction of inflammatory parameters, HE-trained models performed considerably superior to EvG-trained models. Overall, this study represents the first comprehensive evaluation of computational pathology in vascular disease and has the potential to contribute to improved understanding of abdominal aortic aneurysm pathophysiology and personalization of treatment strategies, particularly when integrated with radiological phenotypes and clinical outcomes.

## Introduction

Computational analysis of histopathological imaging has the potential to identify biomarkers, provide insights into disease patterns and pathophysiological processes, and automate histopathological assessment in clinical routine care [1]. The computational evaluation of pathology slides has been studied in several clinical settings, however, most studies have focused on cancer histopathology [2–6]. While the tools of AI in pathology promise to generate new biomarkers and scientific insights, the potential of deep learning (DL)-based approaches in cardiovascular pathology has not been characterized in large patient cohorts to date.

Abdominal aortic aneurysm (AAA) is a pathologic enlargement of the infrarenal aorta with an associated risk of rupture. Pathophysiologically, AAA is considered a degenerative disease of atherosclerotic nature. Many pathological features, such as distinct types of inflammation, angiogenesis in a thickened tunica media, and elastic fiber degradation, including remodeling of the extracellular matrix with different collagen types, are unique to AAA compared to, for example, occlusive disease [7]. Although this connection is debated, increased inflammatory infiltrates may be linked to eventual rupture [8]. Angiogenesis has been demonstrated to be crucial for aneurysm progression, and angiogenesis inhibitors have shown promise for potential non-surgical aneurysm abrogation [9, 10]. Overall, however, despite the clinical significance of AAA, the mechanisms underlying AAA formation and progression remain only partially understood.

We evaluated the performance of DL to identify a range of pathological characteristics including inflammation, fibrosis, elastic fiber degradation, angiogenesis, and calcification from digital whole-slide images (WSI) in an expert-annotated dataset of abdominal aortic aneurysm (AAA) wall samples from 369 patients treated at three European centers. Our work addresses the general lack of generalizable evidence related to the DL-based pathological evaluation of vascular disease samples. Specific to AAA, future extensions of this proof-of-concept will evaluate morphologic features in conjunction with preoperative imaging and patient characteristics and may thereby contribute to the development of imaging biomarkers for AAA progression. Ultimately, such biomarkers could inform personalized treatment strategies and improve clinical outcomes.

## Materials and methods

### Patient cohort

**Fig. 1:**
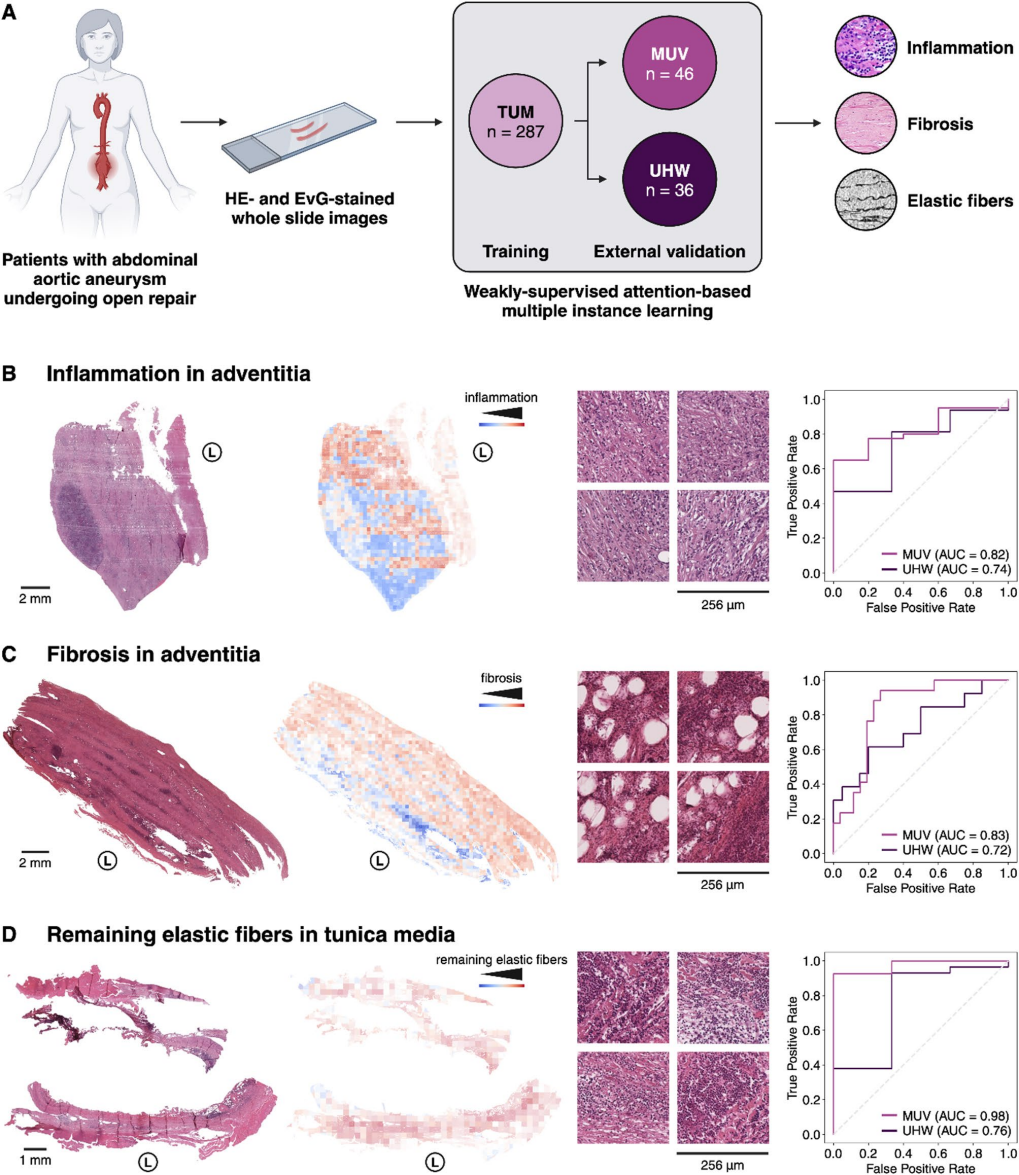
Histopathological evaluation of abdominal aortic aneurysms with deep learning. **(A)** Hematoxylin and Eosin (HE)-stained whole slide images from 369 patients undergoing open aortic aneurysm repair at three European centers were used to develop weakly-supervised deep learning models classifying pathological variables including inflammation **(B)** and fibrosis in the adventitia **(C)**, as well as remaining elastic fibers in the tunica media **(D)**. Model performance was qualitatively evaluated with heatmaps indicating predicted model scores for each 256 μm x 256 μm patch of the whole slide image, as well as with the most predictive patches from the respective slide. Quantitatively, model performance was evaluated using the area under the receiver-operating characteristic curve (AUC) **(B**,** C**,** D)**. Adventitial inflammation **(B)** was classified on a four-category ordinal scale (none, minor, intermediate, major inflammation); the figure displays results from a model differentiating none from the other three classes. Adventitial fibrosis **(C)** was classified on a three-category ordinal scale (minor, intermediate, major fibrosis); the figure displays results from a model differentiating major fibrosis from the other two classes. Remaining elastic fibers in the tunica media **(D)** was classified on a binary scale (< 25%, >25%); the figure displays results from a model differentiating both classes. Abbreviations: Area under the receiver-operating characteristic curve (AUC), Elastica van Gieson (EvG), hematoxylin and eosin (HE), lumen (L), Medical University Vienna (MUV), Technical University Munich (TUM), University Hospital Würzburg (UHW)

Data from a total of 369 patients (84.6% male, mean age 69.1 ± 8.0 years, average maximum diameter 62.9 ± 15.7 mm) undergoing open AAA repair at the Technical University Munich (TUM, *n* = 287), the University Hospital Würzburg (UHW, *n* = 36) and the Medical University Vienna (MUV, *n* = 46), between 2005 and 2019, were included in this study (Fig. 1A). Inclusion criteria were the availability of a full-thickness sample from the left anterior to midline anterior wall of the AAA sac during open aortic repair, enabling detailed histologic analysis, as well as the availability of corresponding disease-related and patient-related clinical data. All participating biobanks were screened retrospectively for patients meeting these inclusion criteria. Indications for open repair were surgical reasons, patient will, or clinician’s choice, in line with international treatment guidelines. Exclusion criteria included patients undergoing endovascular repair and samples not meeting the abovementioned requirements.

### Histopathologic analysis and patient data

Aneurysm samples from the left anterior wall were independently analyzed by three pathologists as described previously [11] to create ground truth data for model training and testing. The following histopathological parameters were evaluated:

- **Intima**: The Histological Inflammation Scale of Aneurysms (HISA) grade [0, 1, 2, 3, 4] was determined for all samples [12].
- **Media**: The media was scored with regard to the presence or absence of calcification [present, absent], the degree of inflammation [none, minor, intermediate, major], presence of neoangiogenesis [present, absent], and the percentage of intact elastic fibers [< 25%, >25%].
- **Adventitia**: Adventitial features were scored with regard to the degree of inflammation [none, minor, intermediate, major] and the degree of fibrosis [minor, intermediate, major]. In addition, the type of inflammatory cells in the adventitia and media were annotated [none, acute, chronic].

Degrees of inflammation and fibrosis were defined as follows:

- **Inflammation**: None: no or only singular inflammatory cells; minor: localized small infiltrates; intermediate: localized and diffuse infiltrates; major: diffuse dense infiltrates.
- **Fibrosis**: Minor: up to a third of visible adventitia with collagenous fiber proliferation; intermediate: up to a half of visible adventitia with collagenous fiber proliferation; major: more than half of visible adventitia with collagenous fiber proliferation.

Each sample was independently scored by two pathologists. In case of disagreement, consensus was reached through discussion.

Hematoxylin and Eosin (HE)- and Elastica van Gieson (EvG)-stained slides were digitized using an Aperio AT2 (Leica, Wetzlar, Germany) slide scanner. Patient age, sex, smoking history, and maximum AAA diameter were gathered as routine clinical parameters.

### Computational models

Weakly-supervised attention-based multiple instance learning models were trained to identify pathological characteristics from HE- and EvG-stained WSI. All models were trained and internally validated by 5-fold cross validation on the TUM cohort and separately tested on external cohorts from MUV and UHW.

Our model architecture consists of a feature extractor based on a pre-trained convolutional neural network (CNN), followed by an attention mechanism that assigns weights to each patch, and a classifier that integrates the weighted features to produce the final prediction. The STAMP protocol was utilized to process the WSIs, as described previously [13]. In brief, WSIs were preprocessed by tessellation into 224 × 224 pixel patches at a magnification of 256 μm per pixel, followed by computational background rejection, and color normalization of HE-stained slides [14]. EvG-stained slides were not color-normalized as no color normalization protocols presently exist for this staining. Subsequently, features were extracted using a pre-trained histology image encoder [15]. To aggregate patches for slide-level predictions, downstream classification tasks were modeled through weakly-supervised attention-based multiple instance learning [6, 16], enabling the correlation of slide-level labels with tissue morphology (Fig. 1A).

Model performance was quantified using the mean area under the receiver operating characteristic (AUC) and standard deviation across 5 cross-validation folds. High-resolution attention heatmaps for qualitative evaluation were generated through a convolutional equivalent of the attMIL model [16].

### Data availability

Due to data privacy regulations, the raw imaging data cannot be published along with this work. The feature vectors extracted from the histopathological WSI and the corresponding clinical endpoints analyzed in this work are available at 10.5281/zenodo.10998463.

### Code availability

Code for preprocessing is available at https://github.com/KatherLab/STAMP. Code for modeling is available at https://github.com/KatherLab/marugoto. Code for spatial heatmaps and top-attention tiles is available at https://github.com/KatherLab/highres-WSI-heatmaps/tree/AAA_heatmaps.

## Results

This analysis included excised AAA samples from 369 patients from three clinical centers. Table 1 indicates variations in patient characteristics for the three contributing centers.

**Table 1:** Patient characteristics. One-way ANOVA was used for groupwise comparison of numerical variables (age and maximum aneurysm diameter). For groupwise comparison of categorical variables, Chi-squared test was conducted. Abbreviations: SD (standard deviation)

Parameter	Metric	**Munich**	Würzburg (external test cohort)	**Vienna**	*p*-value
		(training cohort)		(external test cohort)	
**Age** [years]	mean ± SD	69.1 ± 8.3	70.3 ± 8.2	68.3 ± 5.4	0.56
**Sex**	n (%)				0.45
Male		240 (80.4)	33 (90.9)	39 (82.1)	
Female		47 (19.6)	3 (9.1)	7 (17.9)	
**Smoking history**	n (%)				0.46
Yes		259 (89.2)	32 (87.5)	44 (95.4)	
No		28 (10.8)	4 (12.5)	2 (4.6)	
**Maximum aneurysm diameter** [mm]	mean ± SD	62.8 ± 15.7	67.6 ± 18.0	59.8 ± 13.1	0.08

Using the expert-labeled histopathological AAA specimens, we trained DL models to identify histopathological parameters directly from WSI.

With regard to inflammatory characteristics, DL could reliably predict presence (binary classification, AUC_MUV_ = 0.75 ± 0.10, AUC_UHW_ = 0.85 ± 0.08) and grade of inflammation (multi-class classification, AUC_MUV_ = 0.73 ± 0.10, AUC_UHW_ = 0.78 ± 0.10) in the adventitia from HE-stained slides (Fig. 1B; Table 2). While the models did not include any regional information, areas that were most relevant for prediction of inflammation in the adventitia were indeed localized in the adventitial region of specimens and displayed inflammatory cell infiltration from various cell types (i.e. plasma cells, lymphocytes, Fig. 1B). In contrast, prediction models for presence and grade of inflammation in the tunica media as well as inflammation type in the adventitia and tunica media yielded random prediction results (AUC overlapping 0.50) (Table 2).

With regard to other pathological characteristics, DL could predict the grade of fibrosis in the adventitia (major vs. other, AUC_MUV_ = 0.83 ± 0.02, AUC_UHW_ = 0.72 ± 0.08, Fig. 1C; Table 2) and remaining elastic fibers in the tunica media (AUC_MUV_ = 0.97 ± 0.02, AUC_UHW_ = 0.75 ± 0.12, Fig. 1D; Table 2). Furthermore, DL could differentiate HISA [12] classes (AUC_MUV_ = 0.79 ± 0.10, AUC_UHW_ = 0.77 ± 0.06), historically classified based on the extent of mild and chronic inflammation, lymphocytic infiltration, and fibrosis. Angiogenesis, calcification, sex, and smoking history could not be predicted from HE-stained WSI (AUC overlapping 0.50, Table 2).

Models trained on EvG-stained WSI overall performed similar to models trained on HE-stained WSI for detection of calcification (HE: AUC_MUV_ = 0.50 ± 0.08, AUC_UHW_ = 0.56 ± 0.14; EvG: AUC_MUV_ = 0.40 ± 0.16, AUC_UHW_ = 0.51 ± 0.12), fibrosis (e.g., major fibrosis in the adventitia, HE: AUC_MUV_ = 0.83 ± 0.02, AUC_UHW_ = 0.72 ± 0.08; EvG: AUC_MUV_ = 0.84 ± 0.04, AUC_UHW_ = 0.79 ± 0.07), HISA grade (HE: AUC_MUV_ = 0.79 ± 0.10, AUC_UHW_ = 0.77 ± 0.06; EvG: AUC_MUV_ = 0.78 ± 0.04, AUC_UHW_ = 0.70 ± 0.06), sex (HE: AUC_MUV_ = 0.54 ± 0.14, AUC_UHW_ = 0.54 ± 0.25; EvG: AUC_MUV_ = 0.40 ± 0.14, AUC_UHW_ = 0.42 ± 0.19), and smoking history (HE: AUC_MUV_ = 0.35 ± 0.25, AUC_UHW_ = 0.49 ± 0.14; EvG: AUC_MUV_ = 0.21 ± 0.21, AUC_UHW_ = 0.44 ± 0.20, Table 2). For prediction of the presence (binary classification, HE: AUC_MUV_ = 0.75 ± 0.10, AUC_UHW_ = 0.85 ± 0.08; EvG: AUC_MUV_ = 0.61 ± 0.12, AUC_UHW_ = 0.54 ± 0.11) and grade of inflammation (multi-class classification, HE: AUC_MUV_ = 0.73 ± 0.10, AUC_UHW_ = 0.78 ± 0.10; EvG: AUC_MUV_ = 0.57 ± 0.21, AUC_UHW_ = 0.58 ± 0.11) in the adventitia, HE-trained models performed considerably superior to EvG-trained models. These results suggest that specific staining methods like EvG, in the context of computational pathology of AAA, may not confer substantial additional information as compared to routine HE staining.

**Table 2:** Performance of attention-based multiple instance learning models for histopathological evaluation of abdominal aortic aneurysms. The table indicates the area under the receiver-operating characteristic curve (AUC) values for each prediction target in the two external test cohorts. Models were implemented separately for hematoxylin and Eosin (HE)-stained and Elastica Van Gieson (EvG)-stained whole slide images. Abbreviation: histological inflammation scale of aneurysms (HISA). NA indicates prediction targets that were not analyzed due to a lack of data in the respective cohorts. Specifically, angiogenesis in the media could not be analyzed for the Vienna test cohort due to all samples being negative for neoangiogenesis in this cohort. For the grade of inflammation in the media, no samples were classified as “major inflammation” for patients in the Vienna and Würzburg external test cohorts.

Prediction Target	Hematoxylin & Eosin		Elastica van Gieson	
	Vienna	Würzburg (external test cohort)	Vienna	Würzburg
	(external test cohort)		(external test cohort)	(external test cohort)
**Grade of inflammation in media**				
[none, minor, intermediate, major]				
Overall (multi-class classification)	0.52 ± 0.06	0.55 ± 0.07	0.45 ± 0.06	0.65 ± 0.10
None vs. other	0.52 ± 0.22	0.59 ± 0.15	0.39 ± 0.13	0.64 ± 0.07
Minor vs. other	0.52 ± 0.14	0.54 ± 0.10	0.53 ± 0.17	0.60 ± 0.02
Intermediate vs. other	0.49 ± 0.18	0.56 ± 0.35	0.49 ± 0.11	0.85 ± 0.22
Major vs. other	NA	NA	NA	NA
**Grade of inflammation in adventitia**				
[none, minor, intermediate, major]				
Overall (multi-class classification)	0.73 ± 0.10	0.78 ± 0.10	0.57 ± 0.21	0.58 ± 0.11
None vs. other	0.75 ± 0.10	0.85 ± 0.08	0.61 ± 0.12	0.54 ± 0.11
Minor vs. other	0.56 ± 0.10	0.72 ± 0.14	0.63 ± 0.05	0.59 ± 0.18
Intermediate vs. other	0.65 ± 0.04	0.56 ± 0.06	0.60 ± 0.04	0.63 ± 0.10
Major vs. other	0.84 ± 0.13	0.70 ± 0.11	0.98 ± 0.01	0.73 ± 0.06
**Type of inflammation in adventitia and media**				
[none, acute, chronic]				
None vs. other				
Acute vs. other	0.71 ± 0.13	0.73 ± 0.15	0.62 ± 0.11	0.90 ± 0.11
Chronic vs. other	0.48 ± 0.15	0.59 ± 0.07	0.56 ± 0.07	0.44 ± 0.12
	0.54 ± 0.24	0.50 ± 0.10	0.52 ± 0.07	0.48 ± 0.07
**Angiogenesis in media**	NA	0.46 ± 0.10	NA	0.49 ± 0.14
[present, absent]				
**Calcification in media**	0.50 ± 0.08	0.56 ± 0.14	0.40 ± 0.16	0.51 ± 0.12
[present, absent]				
**Grade of fibrosis in adventitia**				
[minor, intermediate, major]				
Minor vs. other	0.68 ± 0.02	0.79 ± 0.02	0.70 ± 0.06	0.76 ± 0.06
Intermediate vs. other	0.58 ± 0.08	0.57 ± 0.04	0.64 ± 0.10	0.52 ± 0.13
Major vs. other	0.83 ± 0.02	0.72 ± 0.08	0.84 ± 0.04	0.79 ± 0.07
**Remaining elastica in media**	0.97 ± 0.02	0.75 ± 0.12	0.87 ± 0.18	0.67 ± 0.19
[< 25%, >25%]				
**HISA score**				
[0, 1, 2, 3, 4]				
Overall (multi-class classification)	0.79 ± 0.10	0.77 ± 0.06	0.78 ± 0.04	0.70 ± 0.06
**Sex**	0.54 ± 0.14	0.54 ± 0.25	0.40 ± 0.14	0.42 ± 0.19
[male, female]				
**Smoking**	0.35 ± 0.25	0.49 ± 0.14	0.21 ± 0.21	0.44 ± 0.20
[yes, no]				

## Discussion

This study presents the largest evaluation of computational pathology in the context of vascular disease to date. Our results provide evidence that DL can reliably predict histopathological features like inflammation, elastic fiber degradation, and fibrosis from HE-stained AAA pathology slides. In addition, DL can predict the HISA grade from unlabeled pathology, affirming the relevance of this pathological classification established in 1994 [12] and the reproducibility of inflammatory characteristics in histologic examination of AAA.

Prior works in computational vascular histopathology have evaluated basic segmentation tasks in smaller, single-center patient cohorts [17, 18]. Previous DL-based studies in AAA have predominantly focused on aneurysm detection and segmentation or prediction of clinical endpoints such as aneurysm growth or rupture risk from radiological or biomechanical data [19, 20]. In the present work, DL models were trained on single-center histopathological data from 287 patients and were separately tested on data from two external institutions comprising 36 and 46 patients, respectively. For some prediction targets, including the percentage of intact elastic fibers in the tunica media and (to a minor extent) the grades of inflammation and fibrosis in the adventitia, we observed DL performance differences between the two external test sets. These differences may, on the one hand, be attributable to the relatively small sample sizes in the two test sets. On the other hand, it is conceivable that variations in maximum aneurysm diameter and, consequently, differences in strain and remodeling of the aortic wall may contribute to DL performance variability in detecting intact elastic fibers. Overall, these observations underscore the importance of considering demographic and clinical factors when interpreting computational pathology model performance.

Furthermore, our analyses provide insights regarding different staining techniques in computational pathology. While EvG staining is traditionally valuable for evaluating elastic fiber degradation, our deep learning models for analysis of this characteristic trained on the respectively stained samples showed comparable performances when analyzing HE- and EvG-stained slides. This observation suggests that HE staining might be sufficient for computational pathology approaches in AAA, a finding that is in line with recent studies that underscore the value of HE-stained slides in other computational pathology use cases [21, 22]. Variations in staining protocols, such as incubation times and reagent concentrations, may lead to differences in staining intensity and specificity [23, 24]. To account for these differences, we conducted color normalization for the HE-stained WSI. However, no such color normalization techniques are available for specific stainings such as EvG, which may result in larger de-facto inter-institutional variability in the EvG-stained data analyzed in this study. These factors may additionally contribute to the observed performance differences of the weakly supervised DL models on HE- and EvG-stained samples across different prediction targets.

The limitations of our study are primarily related to its proof-of-concept character and retrospective design. First, despite the large number of patients included in this study, not all histopathological and clinical parameters could be analyzed across both test sets. Second, pathological characteristics that are visually discernible for experts were selected as prediction targets. The descriptive character of these prediction targets does not facilitate a detailed interpretation of pathophysiological mechanisms. Third, given the nature of AAA and the impracticability of aortic sampling, our findings cannot provide directly actionable clinical insights. However, our results could eventually contribute to improved understanding of AAA biology and treatment, especially when set in relation to other data modalities (i.e., genomic profiling, radiological imaging, clinical risk factors) in multimodal prediction models [25, 26].

Our finding of computationally discernible inflammatory patterns in the adventitia implies potential for non-invasive diagnostic approaches: Imaging techniques like PET, SPECT, and radiolabeled white blood cell scintigraphy can non-invasively assess inflammation levels in the adventitia [27] and could potentially identify patients who could benefit from pharmacotherapy targeting inflammation [28]. Likewise, modern noninvasive imaging biomarkers could facilitate more precise monitoring of aneurysm progression and identification of patients requiring more frequent follow-up [29]. To maximize translational impact, our work warrants linking of AAA histopathological phenotypes with respective radiological and biomechanical phenotypes as well as clinical outcomes.

## Data Availability

Due to data privacy regulations, the raw imaging data cannot be published along with this work. The feature vectors extracted from the histopathological WSI and the corresponding clinical endpoints analyzed in this work are available at https://doi.org/10.5281/zenodo.10998463. Code for preprocessing is available at https://github.com/KatherLab/STAMP. Code for modeling is available at https://github.com/KatherLab/marugoto. Code for spatial heatmaps and top-attention tiles is available at https://github.com/KatherLab/highres-WSI-heatmaps/tree/AAA_heatmaps.

## Abbreviations

AAA: Abdominal Aortic Aneurysm
AUC: Area under the receiver operating characteristic
EvG: Elastica van Gieson
HE: Hematoxylin and Eosin
HISA: Histological Inflammation Scale of Aneurysms
MUV: Medical University Vienna
TUM: Technical University Munich
UHW: University Hospital Würzburg
WSI: Whole-slide image
